# Phase II multicentre double-blind randomised controlled trial of a Bivalent VaccInation against Salmonella Typhi and Paratyphi A (BiVISTA) using a controlled human infection model of paratyphoid A infection: study protocol

**DOI:** 10.1136/bmjopen-2025-107608

**Published:** 2026-01-27

**Authors:** Margarete Paganotti Vicentine, Naina McCann, Oisin Hennigan, Noshi Maria, Claudia I Juarez Molina, Stanislava Koleva, Mohammod K Islam, Elizabeth Jones, Amy Flaxman, Nicole Day, Allison MacDonald, Mehreen Adnan, Nisha Singh, Sophie Vernon, Eleanor Wilson, Anirudha Vyankatesh Potey, Abhijeet Dharmadhikari, Sanket Gaidhane, Prasad S Kulkarni, Hannah Robinson, Parvinder Aley, Young Chan Kim, Brian Angus, Xinxue Liu, Maheshi Nirmala Ramasamy, Andrew J Pollard

**Affiliations:** 1 University of Oxford Oxford Vaccine Group, Oxford, UK; 2 NIHR Oxford Biomedical Research Centre, Oxford University Hospitals NHS Foundation Trust, Oxford, UK; 3 Oxford Health NHS Foundation Trust, Oxford, UK; 4 University of Oxford, Oxford, UK; 5 Texas Department of Public Safety, Austin, Texas, USA; 6 Serum Institute of India Private Limited, Pune, India; 7 Oxford University Hospitals NHS Foundation Trust, Oxford, UK

**Keywords:** Clinical Trial, Randomized Controlled Trial, Vaccination, Typhoid-Paratyphoid Vaccines, Vaccines, Conjugate, Vaccines, Combined, Vaccine Efficacy, Immunogenicity, Vaccine, Adults

## Abstract

**Introduction:**

Enteric fever, primarily caused by *Salmonella enterica* Typhi and *Salmonella enterica* Paratyphi A (SPA), is endemic mainly in South Asia, disproportionately affecting school-age children. Although typhoid conjugate vaccines (TCVs) are effective and implemented in many countries, no licensed vaccine exists against paratyphoid A. Bivalent vaccines targeting both S. Typhi and SPA may address this gap. Although field efficacy trials are not considered feasible, controlled human infection models (CHIMs) offer an alternative pathway for evaluating vaccine efficacy. This will be the first efficacy study of a bivalent vaccine against typhoid and paratyphoid A using a paratyphoid CHIM.

**Methods and analysis:**

This is a phase II multicentre, double-blind, randomised controlled trial assessing the efficacy and immunogenicity of a bivalent conjugate vaccine candidate, Serum Institute of India Typhoid Conjugate Vaccine (Bivalent) (SII-TCV(B)), against SPA using a CHIM in healthy UK adults aged 18–55 years. A total of 192 participants will be randomised 1:1 to receive either SII-TCV(B) or a licensed Vi-polysaccharide typhoid vaccine (Vi-PS). All participants will be orally challenged with S. Paratyphi A (strain NVGH308) 28 days postvaccination. Participants will be monitored closely for 14 days and treated at 14 days postchallenge or promptly on diagnosis, according to prespecified criteria. The primary objective is to evaluate vaccine efficacy of SII-TCV(B) against paratyphoid infection using a CHIM. The coprimary immunogenicity objective is to assess non-inferiority of the typhoid IgG response compared with a licensed Vi-PS control.

**Ethics and dissemination:**

The study has received ethical approval from the Berkshire Research Ethics Committee (24/SC/0309) and regulatory approval from the UK Medicines and Healthcare products Regulatory Agency. Results will be disseminated via peer-reviewed publications and scientific meetings.

**Trial registration number:**

ISRCTN65855590.

**STRENGTHS AND LIMITATIONS OF THIS STUDY**

- The multicentre, randomised, double-blind, controlled design of the study, with stratified allocation, will provide high-quality data.
- The use of a controlled human infection model will enable efficacy assessment of the first bivalent typhoid-paratyphoid A vaccine candidate, since field trials are not considered feasible due to the high cost and large sample size required.
- Rigorous screening procedures, including gallbladder ultrasound, prevention of chronic faecal carriage of *Salmonella* enterica Paratyphi A and enforced infection control precautions, ensure safety of the participants and their close contacts.
- A centralised administration of challenge associated with fully validated centralised immunogenicity assays (including anti-Vi and anti-LPS IgG and IgA for primary and secondary objectives) will provide consistent endpoints.
- The sample size was calculated to provide 90% power to detect a vaccine efficacy higher than 30% and to demonstrate non-inferiority of Serum Institute of India–Typhoid Conjugate Vaccine(B) compared with a plain polysaccharide typhoid vaccine as control.

## Introduction

 The lack of a licensed vaccine against *Salmonella* enterica Paratyphi A (SPA) is a major gap in enteric fever prevention. Although typhoid fever accounts for the majority of enteric fever cases globally, paratyphoid fever is responsible for over 20% of all cases of enteric fever.^1^ SPA is estimated to be responsible for up to 99% of cases of paratyphoid fever^2 3^ and poses a significant and growing public health threat in low-income and middle-income countries where safe water and sanitation infrastructure are inadequate. With a global annual burden of 14 000 deaths and over 1 million disability-adjusted life years (DALYs) in 2021^4^ attributed to paratyphoid fever alone, it affects predominantly school-age children in South Asia. The epidemiology of paratyphoid disease is evolving, including escalating antimicrobial resistance^5^,^7^ and an increasing proportion of enteric fever cases,^8^ highlighting the need for urgent preventive measures. Although the provision of safe water, sanitation and hygiene measures is urgently needed to prevent enteric fever,^9^,^12^ these take time and financial investment. Vaccines are, therefore, a useful intervention to reduce the paratyphoid fever burden in the meantime.

Historical inactivated whole-cell vaccines against typhoid and paratyphoid A and B were available by the beginning of the 20th century. However, their efficacy against paratyphoid was unclear, and they were discontinued due to unacceptable tolerability.^13 14^ Since then, the development of vaccines against typhoid fever has been prioritised over the investigation of vaccines against paratyphoid fever. Recent efforts to investigate paratyphoid vaccines have concentrated on oral live attenuated^15^ or inactivated vaccines based on O2:LPS antigen,^16^,^18^ and combined vaccines with typhoid and even with other Salmonella enterica serovars are in development.^19^,^23^ However, advancing their development has been challenging due to a lack of an appropriate preclinical animal challenge models (S. Paratyphi A is a human-restricted pathogen) and absence of immunological correlates of protection. Field efficacy trials are considered unaffordable and logistically demanding, requiring as many as over one hundred thousand volunteers, therefore considered infeasible.^24 25^

Licensed vaccines against typhoid fever are available: the monovalent conjugate Vi-polysaccharide vaccines against typhoid (typhoid conjugate vaccines, TCVs) are recommended by WHO since 2017,^26^ considered cost-effective in high-incidence countries^27 28^ and being incorporated into routine immunisation schedules of endemic countries.^29^ TCVs have an efficacy above 80%,^30^,^32^ even in outbreak contexts,^33^ contributing to significant disease prevention since they were administered to over 64 million children.^34^ Although TCVs effectively protect against typhoid fever, there is a gap in the enteric fever prevention, since TCVs do not address the paratyphoid burden and may contribute to a serovar replacement.^35^,^39^

To tackle the enteric fever burden more comprehensively, a vaccine that would extend coverage against paratyphoid as well as typhoid would be desirable for stakeholders.^22 24^ Combined vaccines have multiple advantages over monovalent vaccines. They reduce costs and logistical burdens by reducing materials (eg, syringes, needles), storage requirements and healthcare visits. Additionally, combining typhoid and paratyphoid A vaccines is practical, given their overlapping demographic and geographical distribution in South and Southeast Asia.^40^ Polysaccharide-protein conjugate vaccines have practical advantages over other platforms, including high efficacy even in children as young as 6 months of age due to generation of a T-cell dependent immune response with B-cell memory, alongside low cost and few cold chain requirements.^41^

Licensing new TCVs can be achieved through demonstration of non-inferiority in immunogenicity studies, comparing with licensed Vi-polysaccharide typhoid vaccine (Vi-PS) or other licensed TCVs.^24^ This pathway is not applicable for paratyphoid vaccine candidates. Controlled human infection models (CHIM) provide a cost-efficient and time-efficient alternative to test vaccine efficacy (VE).^24^ The Oxford Vaccine Group (OVG) has established a typhoid^42^ and a paratyphoid CHIM.^43^ The successful experience testing a TCV in this experimental setting^44^ showed the potential of CHIM as a robust tool for testing enteric fever vaccine candidates, providing data towards WHO prequalification of the vaccine.^45 46^ In 2022, the WHO’s Product Development for Vaccines Advisory Committee (PDVAC) suggested CHIMs could be used to accelerate testing and consequent licensure of bivalent typhoid and paratyphoid A vaccines,^24^ and our group has recently evaluated a monovalent paratyphoid VE using CHIM,^47^ results expected in 2025.

This trial will be the first to test a bivalent conjugate vaccine against both typhoidal serovars causing enteric fever using a SPA CHIM. Serum Institute of India Typhoid Conjugate Vaccine (Bivalent) (SII-TCV(B)) was proven safe in a phase I trial^18^ and, if proven efficacious, this vaccine could achieve expedited licensure and prequalification,^24^ allowing comprehensive deployment to tackle the dual burden of typhoid and paratyphoid fever, a significant step forward in global health.

## Study aims and objectives

This study aims to assess the efficacy of one dose of the bivalent conjugate vaccine candidate SII-TCV(B) against SPA in a CHIM, and to demonstrate non-inferiority of the immune response to the S. Typhi component compared with a UK-licensed typhoid vaccine. Secondary and exploratory objectives include characterisation of immune response to the vaccine and to the challenge, assessment of vaccine safety, and investigation of correlates of protection against SPA. Table 1 lists primary and secondary objectives and outcomes, with a complete list found in more detail in the online supplemental table S1.

**Table 1:** Primary and secondary objectives and outcomes of the BiVISTA study

Study objectives	Outcome measures
Primary objectives	
To evaluate the efficacy of SII-TCV(B) against SPA compared with a licensed Vi polysaccharide vaccine in a healthy adult paratyphoid A challenge model	Proportion of participants developing S. Paratyphi A infection within 14 days following challenge, according to pre-specified diagnostic criteria
To evaluate the non-inferiority of the immune response to the *Salmonella* Typhi component of SII-TCV(B) compared with the licensed Vi Polysaccharide vaccine	Geometric mean concentration (GMC) of S. Typhi Vi antigen-specific IgG at day 28 following vaccination
Secondary objectives	
To evaluate the safety and tolerability of SII-TCV(B) compared with a licensed Vi polysaccharide vaccine	Occurrence of: local solicited events in 7 days following vaccination systemic solicited events in 7 days following vaccination unsolicited events in 28 days following vaccination serious adverse events following vaccination throughout study participation
To evaluate the immunogenicity of SII-TCV(B) compared with a licensed Vi polysaccharide vaccine	Quantification of S. Paratyphi A antigen-specific antibodies at 28 days following vaccination: GMCs, geometric mean fold rise (GMFR) and seroconversion (fourfold rise to prevaccination) for anti-LPS IgG, IgA and IgM and SBA titres Quantification of S. Typhi antigen-specific antibodies at 28 days following vaccination: GMCs, GMFR and seroconversion (four-fold rise to prevaccination) for anti-Vi IgG and IgA

BiVISTA, Bivalent Vaccination against *Salmonella* Typhi and SPA; SBA, serum bactericidal assay; SII-TCV(B), Serum Institute of India Typhoid Conjugate Vaccine (Bivalent); TCV, typhoid conjugate vaccine.

## Methods

### Study design

This manuscript is written according to protocol version 2.1, dated 29 January 2025. Two independent researchers and members of the DSMC (Data and Safety Monitoring Committee) reviewed and contributed to the protocol prior to finalisation.

The study is a multicentre, double-blinded, randomised, controlled, outpatient trial of the vaccine candidate SII-TCV(B) against typhoid and paratyphoid A, using a CHIM of healthy adult volunteers experimentally infected with SPA. The planned study period is from October 2024 until September 2027, with the first participant recruited in March 2025.

In total, 192 individuals will be randomised to receive a single intramuscular dose of either SII-TCV(B) or a UK-licensed polysaccharide typhoid vaccine (Vi-PS), Typhim Vi. 28 days after the blinded vaccination, all participants will be orally challenged with a single dose of 1–5×10^3^ CFU (colony-forming units) SPA strain NVGH308. The dose was established in a previous study conducted at the OVG to achieve a 55%–75% attack rate (proportion of participants diagnosed with paratyphoid A infection by prespecified endpoint criteria, see table 2) using the same strain.^43^ This dose was later used in two subsequent SPA CHIM studies at the OVG with attack rate within the same range.^47 48^ All participants will be monitored daily after the challenge, and they will all be treated with antibiotics either at the time they reach the diagnosis criteria or at 14 days, whichever will be sooner.

**Table 2:** Summary of inclusion and exclusion criteria (full list, including temporary exclusion, is described in online supplemental material)

Summary of inclusion and exclusion criteria	
Inclusion criteria	Willing and able to give informed consent. Healthy adults aged between 18 and 55 years. Available for all required appointments at the study centre. Ability to comply with all study requirements, including adherence to good personal hygiene and infection control precautions. Agree to allow the study team to access their medical history and immunisation records. Agree to allow their GP (and/or consultant if appropriate) and National Public Health Agency to be notified of participation in the study. Agree to give their close household contacts written information about their involvement in the study, offering them voluntary screening for S. Paratyphi A carriage. Agree to be contactable by mobile phone and to have a 24-hour contact person to allow the study team to speak to them during the 4 weeks postchallenge. Agree to avoid antipyretic/anti-inflammatory medications during the challenge phase until antibiotic treatment. Agree to be registered onto The Over-Volunteering Prevention System.
Exclusion criteria	History of significant organ/system disease that could interfere with study conduct or completion, in the opinion of the study team. Including but not limited to the following: history of any calculi (urinary or biliary), any gastro-intestinal condition leading to chronic diarrhoea or chronic constipation or use of regular medications for gastrointestinal tract, known or suspected drug or alcohol abuse or history of severe or psychotic mental health condition, or history of cancer. Any impairment of immune function, for example, IgA deficiency, auto-immune disease, HIV infection, use of immunosuppressive drugs or radiotherapy. HLA-B27 positive. Moderate or severe depression or anxiety as classified by the Hospital Anxiety and Depression Score at screening or challenge that is deemed clinically significant. Weight less than 50 kg. Presence of implants or prosthetic material. Taking long-term medication that may affect symptom reporting or interpretation of the study results or that may interact with antibiotics used for treatment of paratyphoid A (particularly drugs that could prolong corrected QT interval). Contraindication to antibiotics used in the study. Family history of aneurysmal disease. Participants who are pregnant, lactating or who are unwilling to ensure effective contraception until faecal clearance. Close house contact or occupation involving direct contact with young children or with anyone immunosuppressed (including pregnant individuals), unless willing to be moved to avoid any contact with these individuals. Anyone in Commercial food handling (involving preparing or serving unwrapped foods not subjected to further heating). Detection of any clinically significant abnormal results from screening investigations. Having been resident in an enteric fever endemic country for 6 months or more. Previous confirmed or suspected typhoid or paratyphoid infection or participation in previous enteric fever challenge studies (with ingestion of challenge agent). Receipt of any typhoid vaccination at any time. Prolonged corrected QT interval (>450 ms) or significant clinical abnormality on ECG. Presence of gallbladder abnormalities (eg, calculi, polyps) on ultrasound. Significant blood donation or planned blood donation prior to study vaccination. Evidence of HIV, hepatitis B or hepatitis C infection. Any other medical or social condition that may affect participation in the study.

GP, general practitioner.

**Figure 1:**
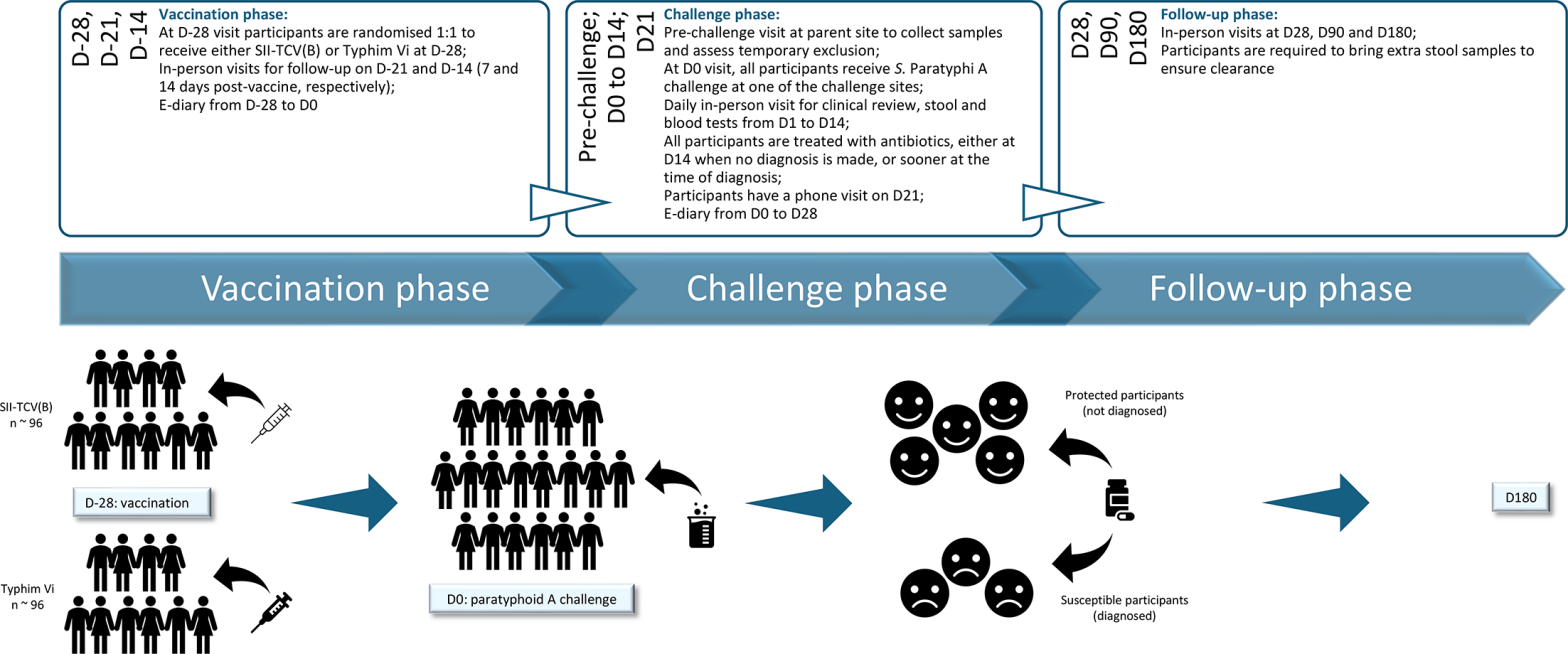
Study design and visit timeline. After screening, participants are vaccinated with intramuscular test or comparator vaccine at D-28, with follow-up visits at D-21 and D-14. All participants undergo oral *Salmonella* enterica Paratyphi A challenge at D0 with daily visits until D14. Antibiotic treatment starts at the time of diagnosis or at day 14 for those who were not diagnosed, whichever is sooner. Participants have a phone follow-up visit at D21 and an in-person follow-up at D28. Electronic diaries collect data from D-28 to D28. After completing treatment, all participants are required to bring three stool samples to ensure bacterial clearance. The last follow-up visits are at D90 and D180. D, day; SII-TCV(B), Serum Institute of India Typhoid Conjugate Vaccine (Bivalent)

The study is sponsored by the University of Oxford and will be conducted in 13 study centres (listed in the online supplemental material). All parent sites are equipped for safe participant review and adequate sample processing. Participants will be recruited, vaccinated and followed up at their parent site. Challenge agent administration will be centralised and conducted under predefined standard operating procedures. A study design diagram is presented in figure 1. Participants will be reimbursed for their time, travel and inconvenience.

Infection prevention and control measures will be in place at the research facilities and taught to participants to practise at home to avoid spread of the challenge strain.

### Recruitment and eligibility

Recruitment advertisements will use approved text and will be widely distributed.

Healthy adult volunteers aged 18–55 years will be directed to read the Participant Information Sheet to undergo a screening process, summarised in online supplemental figure S1, when they will sign an informed consent form, which is available as online supplemental material. Inclusion and exclusion criteria are summarised in table 2 (full list is in online supplemental material). When found eligible, participants will be invited to trial vaccination. On the vaccination day and on the challenge day, temporary exclusion criteria (listed in online supplemental material) will also be applied. The trial management group will actively monitor the representativeness of our trial population.

### Trial procedures

Vaccination will occur at day 28 visit. Challenge day will mark day 0, followed by 14 daily visits. In-person follow-up time points will be at 7, 14 and 28 days after vaccination and 28, 90 and 180 days after challenge, with a phone follow-up on day 21 postchallenge. The visit schedule is detailed in figure 1.

Since infection caused by SPA is notifiable, UK Public Health authorities will be informed about the conduct of the trial and will be notified of challenged individuals, for which participants will be specifically consented.

#### Randomisation

Participants will be randomised in a 1:1 ratio to receive a single intra-muscular dose of SII-TCV(B) or a Vi-PS (Typhim Vi). A randomisation list, generated by an OVG statistician using random block sizes (2 and 4), will be used to ensure equal target size in both arms. The randomisation will be stratified by parent site and uploaded to a web-based system (Research Electronic Data Capture, REDCap) that automates the allocation.

Unblinded clinical staff will run the randomisation on REDCap just before administering vaccine. The unblinded staff will not be involved in any postvaccination procedures and are independent from the blinded team.

#### Blinding

The double-blind will be maintained from study start until the last participant has completed day 28 postchallenge follow-up visit. Unblinding at an earlier time point may occur if treatment allocation is important for a participant’s clinical management, in case of an adverse reaction. In that situation, the DSMC will guide the emergency unblinding.

No unblinded stakeholders will be involved in the assessment of study participants or in study endpoint data collection or in laboratory analysis. Unblinded members will include the statistician who generates the randomisation schedule, selected data managers and monitors, and the clinical staff responsible for vaccine preparation and administration. Blinding is implemented to minimise the risk of bias.

Both vaccines have distinct appearances. For this reason, to protect the blinding, all vaccine-related procedures will be conducted away from participants and from blinded staff. Syringes will be masked with opaque tape to reduce the risk of inadvertent unblinding.

#### Vaccination visit and vaccination phase

Allocated vaccine will be administered at visit day 28, after confirming that consent is valid and no temporary exclusion criterion applies. Participants will then be observed for a minimum of 45 min and asked to record solicited symptoms (listed in online supplemental tables S3, S4) daily for 7 days and unsolicited symptoms for 28 days on an electronic diary.

Follow-up visits with safety assessment and sampling will occur at day 21 and day 14 (7 and 14 days after vaccination). At 28 days after vaccination, a prechallenge visit will be conducted for blood sampling.

#### Challenge visit and challenge phase

At the challenge site, participants will be given a pre-treatment with sodium bicarbonate solution 1 min before being orally challenged with SPA (strain NVGH308) at the dose of 1–5×10^3^ CFU suspended in 30 mL of sodium bicarbonate on visit D0. Participants will fast for 90 min before and after challenge administration. They will be directly monitored for a minimum of 15 min, but they remain on site for 2 hours; if they vomit within 90 min of challenge administration, they will be withdrawn from the study and treated with antibiotics. During this observation, participants will be instructed and observed practising hand hygiene according to WHO’s guidance. They will be given access to an e-diary, where they will record solicited symptoms daily (as seen in online supplemental table S5) for 21 days, including twice-daily measured temperatures, and they may add any unsolicited symptoms and medications for 28 days.

Box 1 lists the prespecified criteria for diagnosis of paratyphoid infection: if presenting with at least one of them, participants will be considered susceptible to the disease, prompting antibiotic treatment. Recruited participants will be naïve to enteric fever, and baseline stool samples will be collected to ensure no SPA will be present prior to challenge. Since the UK is not endemic for enteric fever, and participants will not travel to endemic countries, we will assume that all SPA recovered from blood or stool culture will be the challenge strain.

Box 1 **Criteria for the diagnosis of paratyphoid A infection**

Paratyphoid A fever is diagnosed if any of the following apply

- A positive blood culture for SPA from 72 hours postchallenge.
- A positive blood culture for S. Paratyphi A within 72 hours postchallenge, with one or more signs/symptoms of paratyphoid A infection (such as recorded oral temperature ≥38.0^o^C).
- Persistent positive blood cultures (two or more blood cultures taken at least 4 hours apart) for S. Paratyphi A within 72 hours postchallenge.
- Oral temperature ≥38.0^o^C persisting for 12 hours or longer, not necessarily continuously.

During the challenge phase, participants will be asked not to take any anti-pyretics to prevent interference with the diagnostic criteria. Participants will be seen daily until day 14, and if clinically indicated, an additional review will be arranged and symptomatic medications prescribed. Participants will be reviewed for 4 days after diagnosis, if applicable. At day 14, all undiagnosed participants will also take a full course of antibiotics.

#### Follow-up visits

After the challenge phase, follow-up visits occur at days 28, 90 and 180 for safety monitoring and for sampling.

To confirm SPA clearance, all participants will bring three faecal samples at least 48 hours apart, at least 1 week after completing antibiotics. When all three samples are culture-negative for SPA, faecal clearance is confirmed. If any sample is positive, the participant will be retreated and followed up until clearance can be confirmed, to ensure no chronic carriage of SPA.

#### Laboratory testing

Samples collected for safety monitoring and microbiological endpoints will be processed and reported locally. For the primary and secondary endpoints, immunological assays to evaluate levels of anti-Vi and anti-LPS IgG, IgA and serum bactericidal assays will be validated and undertaken at an external laboratory. Exploratory endpoints will be undertaken at OVG. Blood sample collection and other samples timepoints are summarised in online supplemental table S7.

Participants will also be able to consent to transfer unused samples to the Oxford Vaccine Centre Biobank at the end of the study.

### Data management

#### Data collection

Clinical data will be collected and recorded directly into electronic case report forms using REDCap. Data entry will be quality-controlled by a second staff member. Laboratory data will be stored securely on University of Oxford’s servers. The OVG team will inspect data collection and storage throughout the study, and an external monitor will oversee monitoring.

#### Confidentiality

The study will comply with Good Clinical Practice, with the UK General Data Protection Regulation and Data Protection Act 2018, and with Sponsor security policies.

### Trial Interventions

#### Vaccine: test arm

SII-TCV(B) is an investigational bivalent conjugate vaccine against S. Typhi and Paratyphi A, manufactured by Serum Institute of India, Pune, India. A single dose of 0.5 mL contains 25 µg of the Vi polysaccharide antigen from S. Typhi conjugated to purified tetanus toxoid and 25 µg of the purified O-specific polysaccharide from S. Paratyphi A (O:2) conjugated to purified diphtheria toxoid. It is administered as a single dose, via intramuscular route. A double-blind Phase I study conducted in 60 adults in India demonstrated SII-TCV(B) to be safe and immunogenic.^18^

Single-dose vial presentation will be used. The clear, colourless to pale yellow liquid will be administered using a syringe with needle in a manner to closely match the comparator presentation.

#### Vaccine: comparator arm

Typhim Vi, a Vi-PS manufactured by Sanofi (Gentilly, France)^49^ and licensed in the UK, will be used as the comparator vaccine. Typhim Vi is indicated for active immunisation against typhoid fever via intramuscular route. A single dose of 0.5 mL contains 25 µg of the Vi-polysaccharide antigen from S. Typhi.

Prefilled single-dose syringe presentation will be used, containing this colourless solution.

#### Challenge

The original SPA strain NVGH308 was isolated in 2006 from a patient in Nepal.^50^ This strain was fully characterised and known to be sensitive to all antimicrobials routinely used to treat enteric fever. Genome sequencing has been completed at the Sanger Institute, Cambridge, UK (labelled ED199). It was manufactured to Good Manufacturing Practice standard by Novartis Vaccines and Diagnostics. The suspension vials have been stored frozen in OVG since then. Ongoing stability tests conducted by GenIbet demonstrate the viability of the challenge agent. This challenge agent has been safely used in three other trials conducted by OVG,^43 47 48^ at a combined attack rate of 66.2% (49/74, 95% CI 54.3% to 76.8%).

The challenge dose will be prepared to contain 1–5×10^3^ CFU in a 30 mL bicarbonate solution for oral ingestion, 1 min after ingestion of 120 mL of a bicarbonate buffer. Final dose is confirmed following overnight incubation in an agar plate.

#### Antibiotics

Treatment will be given to all participants, either at the time of diagnosis or at day 14 postchallenge for those who did not reach preset criteria for diagnosis. Participants will be observed taking the first dose of antibiotic; subsequent doses of antibiotics will be taken by the participant at home, with compliance checked via e-diary. Other reasons for commencing antibiotics are listed in the online supplemental box S1.

A 14-day course of ciprofloxacin (500 mg two times per day) was the first-line antibiotic course used in previous paratyphoid fever CHIMs.^43 47 48^ However, due to potential risks associated with ciprofloxacin, highlighted by the Medicines and Healthcare products Regulatory Agency (MHRA) alert in 2024,^51^ alternative strategies to reduce exposure to this antibiotic were explored. According to the British Infection Association, both azithromycin and ciprofloxacin are recommended as optimal oral options to treat enteric fever.^52^ Evidence from enteric fever CHIMs indicates that ciprofloxacin outperforms azithromycin, with quicker clearance of fever and bacteraemia, as well as quicker symptom resolution,^53^ resulting in improved antibiotic tolerability and significantly fewer instances of antimicrobial switching (unpublished data). To leverage the rapid efficacy of ciprofloxacin while mitigating potential safety concerns and reducing exposure to it, a combination regimen of 4 days of ciprofloxacin (750 mg twice daily) followed by 10 days of azithromycin (1 g on the first day, 500 mg on following days) will be the first-line treatment in this trial.

Second-line or third-line courses of antimicrobials will be offered, should a participant develop intolerance to the first line. Online supplemental box S2 lists the alternatives. In case the participant requires intravenous antibiotics, ceftriaxone will be the choice.

#### Other trial interventions

Other medications will be provided for symptomatic control of enteric fever if required, listed in the online supplemental table S8. No other trial interventions are planned.

### Sample size

The attack rate of the SPA challenge dose was estimated to be 58% on the comparator (Typhim Vi) arm, based on two previous CHIMs (22/38 participants).^43 48^ To show a VE of 70%, corresponding to an attack rate in the SII-TCV(B) arm of 17.4%, the study will need to recruit an effective sample size of 85 per arm. This is necessary to reject the null hypothesis that VE is ≤30% with 90% power at two-sided 0.05 significance level. Accounting for a dropout rate of 10% (although the attrition rate has been lower in enteric fever CHIMs), the final sample size needed is 192 volunteers.

This sample size will provide 90% power to demonstrate non-inferiority of SII-TCV(B) compared with Typhim Vi at a two-sided 0.05 significance level, assuming a 5% attrition rate. A few assumptions were used for the power calculation. First, to determine the non-inferiority of SII-TCV(B), we used WHO recommended non-inferiority margin of 0.67.^54^ Additionally, based on published data, SDs of anti-Vi IgG are 0.57 and 0.47 at log10 scale, respectively, in the Vi-PS and in the SII-TCV(B) arms, with a true geometric mean ratio (GMR) >1.2.^44^

For the primary paratyphoid endpoint (efficacy), the 30% superiority margin probably represents a stringent threshold for a VE trial using CHIM, as demonstrated in prior typhoid vaccine CHIM trials, in which VE was lower than observed in field trials,^44 55^ partly due to stringent predefined diagnostic criteria. Considering this, we conducted a sensitivity power calculation assuming the true VE is as low as 55% in the CHIM and concluded that the current sample size remains robust, providing 80% power to reject the null hypothesis that VE is ≤19% (see table 3).

**Table 3:** Superiority margins at different study powers and associated vaccine efficacy

Vaccine efficacy	Superiority margin (90% power)	Superiority margin (80% power)
50%	8%	14%
55%	13%	19%
60%	19%	24%
65%	24%	30%
70%	30%	35%

### Statistical analysis

The proportion of participants with a diagnosis of paratyphoid fever (the attack rate) in each arm with 95% CI will be presented after window for primary endpoint evaluation (after challenge phase). The VE will be estimated by calculating the difference in attack rate in the comparator and in the SII-TCV(B) arm relative to the attack rate in the comparator arm, as seen below:

 VE=100×(AR_comparator_−AR_SII-TCV(B)_)/AR_comparator_=100×(1−AR_SII-TCV(B)_/ AR_comparator_)

Where VE is the VE and AR is the attack rate. The 95% CI of VE will also be calculated—if the lower boundary of the 95% CI is at or above 30%, the vaccine will be considered efficacious to protect against paratyphoid.

The immunogenicity data will be log-transformed prior to analysis to render a normal distribution, as data are expected to be highly skewed. Values below the limit of detection will be replaced by half the value of the lower limit before transformation. Primary analysis will be conducted on a per-protocol basis for all participants who received vaccination with no critical protocol deviation. A modified intention-to-treat analysis will also be conducted as a sensitivity analysis.^56^

The geometric mean concentration of antibodies will be calculated for each arm as the antilogarithm transformation of the mean of the log10 transformed concentration, with 95% CI calculated as the antilogarithm transformation of the lower and upper limits.

For the non-inferiority primary endpoint, the GMR will be calculated as antilogarithm of the difference between the mean of the log10 transformed concentration in the SII-TCV(B) and in the comparator arms. The 95% CI will be calculated as the antilogarithm transformation of the lower and upper limits. If the lower boundary of the 95% CI of the GMR is above 0.67,^54^ this bivalent vaccine will be considered non-inferior to the comparator Vi-PS.

Secondary analysis will use the Kaplan-Meier method to conduct time-to-event analyses, such as time to positive blood culture or time to oral temperature ≥38 ºC. Participants not meeting criteria for an individual component of the primary endpoint, or who withdrew or had potential interference with vaccine effect or infection challenge, will be censored in the analysis. Sensitivity analyses by sex and age and other variables will be added.

Exploratory objectives will aim to generate new hypotheses; thus, they will be analysed descriptively.

## Ethics and dissemination

### Ethical approval, regulation and governance

The investigators will conduct the study in accordance with relevant regulations and with the Declaration of Helsinki, complying with the Good Clinical Practice ICH E6(R2) and latest versions, where applicable.

Ethical approval was obtained from the Berkshire Research Ethics Committee (REC) (Reference: 24/SC/0309). Health Research Authority and Health and Care Research Wales approved the study. MHRA, which regulates the use of the unlicensed vaccine and the comparator in the study, has authorised the trial. It is registered with ISRCTN under ISRCTN65855590.

Annual reports will be prepared, including a development safety update report.

### Safety and potential risks

There are several potential risks associated with the trial conduct, such as study fatigue, phlebotomy-related complications, vaccine-related adverse events and challenge-associated risks. The study is covered by a clinical trials insurance policy.

Serious adverse events (SAEs), as defined in the online supplemental table S9, will be monitored from the time of enrolment. Any vaccine-related SAE will also be reported as SUSAR (suspected unexpected serious adverse reaction) to all principal investigators, sponsor-delegated investigators, MHRA, REC and other relevant authorities, according to regulations.

If group holding rules, shown in the online supplemental figure S2, are triggered, the study will be paused pending a review by the sponsor and the DSMC, and MHRA and REC will be notified. The decision to terminate the trial or to proceed will be based on the available evidence on the safety of the vaccine. When deemed safe to proceed, a request to the MHRA and Ethics Committee will be submitted, which may include an amendment with changes to the protocol, to the Participant Information Sheet and to the reference safety information of the vaccine Investigator Brochure, as applicable.

The DSMC comprises five independent experienced researchers who will provide real-time safety oversight.

Study fatigue and mood disturbances will be monitored throughout the study with the application of Hospital Anxiety and Depression score questionnaire^57^ at screening, prevaccination and prechallenge visits; additionally, there will be a daily mood question in the challenge diary. Phlebotomy-related complications are rare; they will be monitored clinically and by laboratory results and managed by the study clinicians. Should a participant develop anaemia during this study, sample volumes will be minimised to include only essential safety blood tests, including blood cultures, where appropriate.

Individual participants will be discontinued from the trial in case of a critical protocol deviation, vomiting within 90 min from challenge administration or severe adverse reaction to the vaccine. At least the safety follow-up will be completed until day 180, provided participant consents to it.

#### Vaccine candidate SII-TCV(B)

A phase I controlled randomised study conducted in 60 healthy adults in India demonstrated the safety of the vaccine, with no SAEs and no long-term potential risks to recipients.^18^ Most commonly reported symptoms (injection site pain, headache and myalgia) were all of mild to moderate severity.^18^ To reduce the risk of hypersensitivity, volunteers with a history of allergy or intolerance to any of the vaccine components will be excluded.

Participants will be provided details for contact with a study doctor 24 hours a day, 7 days a week. Additionally, study teams will have participants’ contact details and also details of a 24-hour contact nominated by the participants.

#### Comparator vaccine Typhim Vi

Local reactions such as pain, swelling and redness at the injection site are the most common adverse reactions following this vaccine and are usually mild and transient, based on estimates from clinical trials and postmarketing surveillance data.^49 58^ Rare cases of anaphylaxis after its administration were reported.

#### Challenge

Some participants may develop symptoms of paratyphoid fever during the challenge phase, and there is a potential risk of severe disease. To mitigate risks, common symptoms of enteric fever and its severity will be monitored closely during the challenge phase during visits and via an electronic diary (online supplemental table S5), and severe disease, as defined by prespecified criteria (see box 2), will be monitored. Additional to an in-clinic daily review, the study doctor will review blood results and e-diary entries every evening. Antibiotics will be initiated within 24 hours (typically much sooner) of diagnosis criteria being met or on day 14 postchallenge, whichever occurs first. Due to a mild-moderate symptom profile,^43 48 59^ most participants are expected to be diagnosed with the endpoint of positive blood cultures rather than as a result of persistent fever.

Box 2 **Adverse events for special consideration related to challenge and criteria for severe paratyphoid A disease**

Adverse events for special consideration related to challenge

- Severe paratyphoid A infection, as defined in the protocol:
  - Oral temperature >40.0ºC.
  - Systolic blood pressure <85 mm Hg.
  - Significant lethargy or confusion.
  - Gastrointestinal bleeding.
  - Gastrointestinal perforation.
  - Any grade 4 laboratory abnormality.
- Failure to clinically or bacteriologically cure a participant of paratyphoid A infection: defined as persistent clinical symptoms or persistent bacteraemia within 14 days of start of effective antimicrobial treatment.
- Progression to convalescent shedding of bacteria: defined as positive stool culture at least one week after completion of second course of antibiotics, in line with United Kingdom Health Security Agency (UKHSA) guidance.
- Progression to chronic carrier state: defined as positive stool culture at least one year following challenge.
- Relapse of paratyphoid A infection: defined as recurrence of confirmed paratyphoid A infection following successful treatment.
- Transmission of S. Paratyphi A to a contact of a participant.
- Adverse events requiring a physician visit or emergency department visit which, in the opinion of study staff, are related to the challenge with S. Paratyphi A.

Participants will be informed of the possibility of complications of S. Paratyphi A infection during screening, such as intestinal perforation or haemorrhage. However, this risk is low, as such complications occur almost exclusively in patients who do not receive appropriate treatment for over 2 weeks.^60^

There is a small risk of relapse of infection, defined as recurrence of confirmed paratyphoid infection following successful treatment, typically within 4 weeks of treatment. Participants will be informed of this and advised to contact the study doctor if symptoms recur. Relapse will be managed with a repeat course of antibiotics.

### Antibiotics

Ciprofloxacin and azithromycin, which will be the first line of treatment, are generally well tolerated. However, ciprofloxacin has been associated with rare but potentially serious side effects, including neuropathy, arrhythmia, tendonitis, mood disturbances and aneurysms.^51 61 62^ These risks will be mitigated by reducing the exposure duration and by monitoring possible side effects via e-diary and phone call on day 21. Participants will be advised to contact the study team in case of any side effects.

Screening will exclude volunteers at increased risk of complications, including those with uncontrolled hypertension, depression or anxiety, prior intolerance to antibiotics that may be used in the trial, prolonged QT interval or family history of aneurysm. Counselling will address potential side effects of all antibiotics used, including provision of the MHRA leaflet on the use of ciprofloxacin.^63^ This will happen at screening, on challenge day and at the time of first dose of antimicrobial.

#### Chronic carriage

After treating and recovering from a typhoidal Salmonella infection, individuals may asymptomatically shed these bacilli in faeces. Up to 2%–5% of patients fail to clear the bacteria within 1 year, becoming chronic carriers and contributing to transmission.^64 65^ Over 90% of chronic carriers have underlying gallbladder disease, mainly calculi,^66^ thus only volunteers with a normal ultrasound examination of the gallbladder, performed at screening, will be eligible for inclusion.

The risk of chronic carriage is extremely low in the CHIM.^42 43 48 55^ The classes of the antibiotics used as first line in the trial are the preferred for carriage prevention.^67^,^69^ Participants will be required to bring stool samples after completing antibiotics to ensure clearance of faecal shedding of S. Paratyphi A.

#### Close contacts

Although unlikely, secondary transmission of the challenge strain to household or close contacts may occur via faecal-oral route. To minimise the risk, participants will be required to follow stringent handwashing practices and will receive detailed guidance on hygiene and preventing transmission. Soap and paper towels will be provided. Individuals in high-risk occupations (eg, food handlers and those working with vulnerable populations) will be excluded, unless they agree to abstain from work until clearance of infection is confirmed. Additionally, early treatment provided to participants will ensure rapid bacterial clearance, limiting the excretion period. These measures align with UKHSA guidance to safeguard public health.^70^

### AE reporting

Participants will self-record and grade (0–4) solicited symptoms in the electronic diary for 7 days after vaccination and for 21 days after challenge. Clinicians will regularly review diaries and may contact participants if clarifications are needed. Participants will also be able to record unsolicited symptoms for 28 days from vaccination and challenge, respectively. Solicited symptoms and severity grading of AEs are described in online supplemental tables S2–S6.

AEs related to vital signs recorded in clinic and clinically significant laboratory abnormalities will be reported from vaccination to day 180, whereas SAEs will be reported from consent signing until day 180 postchallenge. Additionally, AEs related to challenge for special consideration (AERC), listed in box 2, will be monitored from challenge day through day 180 postchallenge.

Solicited symptoms will be automatically presumed to be related to the vaccine or to the challenge, depending on the diary in which they were recorded. Clinicians will complete causality assessment for all other AEs, including unsolicited symptoms, throughout the study. Events will be considered related or not related to vaccination, challenge or other interventions (eg, antibiotics).

All SAEs and AERCs will be reported to the sponsor-delegated investigator and DSMC within 24 hours of notification. SAEs related to the vaccine will be reported as SUSAR within the required regulatory timelines.

### Dissemination

The findings will be disseminated through peer-reviewed journals and relevant scientific conferences. All publications will adhere to the Consolidated Standards of Reporting Trials guidelines and the requirements of International Committee of Medical Journal Editors.

### Patient and public involvement

A patient and public involvement and engagement group reviewed participant-facing materials, including information leaflets and recruitment advertisements. Their suggestions were incorporated into these materials to ensure clarity and accessibility for potential participants. This group will also support advertising strategies, recruitment planning and public engagement initiatives.

Additionally, a regular newsletter disseminating study results and updates on ongoing trials will be sent out for individuals who opt to receive them. Social media platforms will also be used to engage followers with real-time updates about the research.

## Discussion

This will be the first CHIM study to evaluate the efficacy of a bivalent vaccine against S. Typhi and Paratyphi A fever. Bivalent vaccines against both serovars would be of particular importance in South Asia, where these pathogens are co-endemic, with overlapping epidemiology, affecting mainly school-age children.^1 4 40 71 72^ Such vaccines would contribute to achieving a more comprehensive control of enteric fever.

The CHIM will enable testing the efficacy of the paratyphoid component, since field trials are not considered feasible, requiring thousands of volunteers and prolonged follow-up.^24^ This aligns with the WHO’s PDVAC suggestion to use CHIMs to evaluate the efficacy of the paratyphoid component of bivalent vaccines.^24^ Testing non-inferiority of the typhoid component was also suggested by PDVAC to allow vaccine licensure.^24^ Although intended for endemic countries market, if proven efficacious, comparison with a typhoid vaccine routinely used in travel clinics may inform regulatory approval for travellers, with protection against paratyphoid A as an added benefit.

To ensure safe conduct of this CHIM, clearly articulated ethical safeguards will be implemented, including close clinical monitoring and prompt initiation of antibiotic therapy at diagnosis or at 14 days in those who do not develop enteric fever to prevent infectious complications. Thorough and comprehensive informed consent, supported by sustained communication with the participants and consistent safety oversight across all study sites, will be key to the responsible conduct of this trial.

Since the CHIM population will differ from those most affected by enteric fever in terms of previous exposure and age,^4^ licensure and deployment to endemic countries would require additional steps. Data generated in this study would require bridging with immunogenicity data^24^ from a planned phase III study in India, where the vaccine will be tested in adults and children. Young children from endemic countries are naïve to paratyphoid, suggesting that protective responses in UK adults found in the CHIM may serve as immunological surrogates. This assumption is based on similar studies with typhoid in endemic areas, where TCV has demonstrated efficacy exceeding 80%.^30^,^33^

Although age differences between these two populations may influence vaccine-induced immune responses, particularly with respect to B-cell maturation, this effect is less pronounced for conjugate vaccines,^73^ which elicit T-cell-dependent antibody production.^74^ Pre-existing memory B-cells from prior exposure in endemic settings may further influence vaccine responses.^73^ Bridging CHIM-derived findings with age-stratified serological data from endemic populations will be essential for contextualising correlations. Notably, antibody levels conferring protection in the typhoid CHIM^44^ have been shown to be comparable to those observed in an endemic setting.^32^

The multicentre double-blind randomised controlled design with stratified allocation will provide high-quality data, with multiple centres facilitating recruitment. The sample size will provide sufficient statistical power to detect both primary endpoints. Although CHIMs may underestimate VE, as observed for TCV,^44^ likely due to stringent diagnostic criteria and large inoculum used to ensure high attack rates, the sample size maintains adequate power to detect a clinically meaningful efficacy. Based on previous studies,^18 44^ the typhoid component is expected to demonstrate non-inferiority compared with the Vi-PS vaccine. This trial will also include blood sampling to investigate correlates of protection against paratyphoid A, which remain unknown and would inform future vaccine development.

Functional antibody assays and assessment of cellular immunity may help elucidate protective responses against S. Paratyphi A infection, as observed with other enteric or Gram-negative pathogens. Antibody-dependent opsonophagocytic activity correlates with vaccine-induced protection for Vi-based typhoid vaccines^75^ and pneumococcal conjugate vaccines.^76^ Antibody-mediated complement bacterial killing serves as a correlate of protection for cholera^76^ and meningococcal vaccines^76 77^ and is associated with reduced disease severity post live typhoid vaccines^78^ and post shigella infection in a CHIM.^79^ IgA responses, mucosal immunity and memory B cells also contribute meaningfully to protection against shigellosis^80 81^ and typhoid infection.^75 81^

If this bivalent vaccine demonstrates efficacy against S. Paratyphi A, it will provide more comprehensive protection against enteric fever than TCVs. Data from this CHIM combined with the phase III study in India would support licensure in India and could be used toward WHO prequalification. The CHIM was pivotal to the process that led to the TCV prequalification by WHO^44^,^46^ and facilitated investigation of immunological correlates of protection against typhoid fever.^7582^,^84^

While long-term solutions must prioritise equitable access to safe water, sanitation and hygiene,^11^ they take time. Deployment of an effective bivalent vaccine would alleviate the burden from both pathogens, reduce strain on healthcare systems in endemic regions and potentially contribute to slowing antimicrobial resistance spread.^33 85^

## Supplementary material

10.1136/bmjopen-2025-107608 online supplemental file 1

10.1136/bmjopen-2025-107608 online supplemental file 2
