# Supplementary material for: Phase II multicentre double-blind randomised controlled trial of a Bivalent VaccInation against Salmonella Typhi and Paratyphi A (BiVISTA) using a controlled human infection model of paratyphoid A infection: study protocol
Source: BMJ Open. 2026 Jan 27;16(1):e107608. doi: 10.1136/bmjopen-2025-107608 (PMC12853460; doi:10.1136/bmjopen-2025-107608)
Supplement: online supplemental file 1 [file bmjopen-16-1-s001.pdf]

BiVISTA
Bivalent Vaccination against *Salmonella* Typhi and Paratyphi A

CONSENT FORM

Participant Number: BIV- 01 __ __ __ __ __ __

Participant Name: ______________________________

Participant Initials: ________

If you agree, please INITIAL box:

**Section 1: Study Procedures**	
1. I confirm that I have read and understood the participant information sheet Version: .... Dated........................ for this study. I have had the opportunity to consider the information, ask questions and have had these answered satisfactorily.	
2. I have received detailed information about the treatment schedule and potential side effects and have discussed any potential questions.	
3. I understand that my participation is voluntary and that I'm free to withdraw at any time without giving any reasons, without my medical care or legal rights being affected.	
4. Should I wish to withdraw after I have been challenged with *Salmonella* Paratyphi A, I understand that I must take a course of antibiotics and will be asked to attend for further visits for safety reasons and that if I fail to do so United Kingdom Health Security Agency (UKHSA)/Public Health Scotland/Public Health Wales will be informed.	
5. I understand that should I fail to return for review or to take the full course of antibiotics I may become seriously ill and could even die.	
6. I will bring the 24-hour contact reply slip to the first study visit, signed by my 24-hour contact. I agree that the study team may contact this person if I cannot be contacted during the study.	
7. I agree to refrain from donating blood for the duration of the study.	

Participant Number: BIV- 01 __ __ __ __ __ __

**Section 2: Personal Information:**	
8. I agree to my GP being informed of my participation in this study including information about diagnosis, treatment and clearance samples.	
9. I agree to my GP and/or other treating doctors being approached for additional information regarding my medical and vaccination history, if pertinent to this study.	
10. I understand that United Kingdom Health Security Agency (UKHSA)/Public Health Scotland/Public Health Wales will be informed of my participation in this study including information about diagnosis, treatment, and clearance samples.	
11. I understand that relevant sections of my medical notes and data collected during the study including identifiable information may be looked at by individuals from [INSERT SITE(S)], from regulatory authorities, from the NHS Trust(s), appointed UK private health laboratories if applicable and authorised representatives appointed by the Sponsor (University of Oxford), where it is relevant to my taking part in this research. I give permission for these individuals to have access to my records.	
12. I understand that data collected during the study including information from which I may be able to be identified may be shared with the study funder (Serum Institute of India) and regulators as needed for potential future licensing of the SII TCV(B) vaccine.	
13. I understand TOPS is the Health Research Authority database that aims to prevent healthy volunteers from taking part in too many studies. I understand that only staff at [INSERT SITE NAME] and other research units can use the database, and the study team will check volunteer details. I agree to my National Insurance (if UK citizen) and Passport number (for non-UK citizens) being used to register me on TOPS. I understand that it will be stored electronically for the duration of the study.	
14. I understand and agree that the study data, including identifiable information, will be held securely on a server at the University of Oxford.	
**Section 3: Research Samples**	
15. I agree to donate saliva [if applicable], blood and stool samples. I consider these samples a gift to the University of Oxford, and I understand I will not gain any direct personal or financial benefit from them.	
16. I understand that my data and biological samples may be sent and stored within and outside of the United Kingdom for analysis by collaborating research groups and laboratories as described in the participant information sheet.	

Participant Number: BIV- 01 __ __ __ __ __ __

17. I agree to have blood tests as part of this study, including testing for HIV, Hepatitis B and Hepatitis C. I agree to results of Hepatitis B and C blood tests conducted as part of this study being reported to the United Kingdom Health Security Agency (UKHSA)/Public Health Scotland/Public Health Wales as outlined in the participant information sheet if required.	
18. I understand and agree that my samples will be used in research aimed at understanding the genetic influences of response to vaccination against, and infection with, the bacteria that causes paratyphoid A disease and that the results of these investigations are unlikely to have any implications for me personally.	
**Section 4: Occupational**	
19. I understand that I should not be involved in commercial food handling until I am shown not to be infected with *Salmonella* Paratyphi A	
20. I understand my occupation must not involve direct contact with young children (defined as those attending pre-school groups or nursery or aged under 2 years) or patient contact in a health or social care setting until I am shown not to be infected with *Salmonella* Paratyphi A	
**Section 5: Additional**:	
21. I understand I should not have household contacts who are immunocompromised (including pregnancy) or young children (under 2 years or attending pre-school or nursery)	
**Section 6:** ***Questions 22 and 23 may not apply to you: please INITIAL top box if it applies to you or INITIAL the N/A box accordingly if it does not apply to you.***	
22. **For those involved in the provision of health or social care to vulnerable groups only:** I agree to my employer being informed of my participation in the study.	 N/A
23. **Participants of childbearing potential only:** I understand the need to ensure that I or my partner use effective contraception one month prior to vaccination and continue to do so until I am shown not to be infected with *Salmonella* Paratyphi A. I also understand that if I use oral hormonal contraception there is a need to use barrier contraception from vaccination until I am shown to be cleared of infection.	 N/A
**Section 7: SUMMARY**	
24. I agree to take part in this study.	

Participant Number: BIV- 01 __ __ __ __ __ __

**Section 8:** ***OPTIONAL (please INITIAL box yes or no accordingly):***		
25. I agree to be contacted about other ethically approved research studies for which I may be suitable. I understand that agreeing to be contacted does not oblige me to participate in any further studies.	Yes	No
26. I agree for my samples to be used, in a form that does not identify me, in future research here or abroad, which has ethics approval. I understand this research may involve commercial organisations.	Yes	No

_______________________ __ __ / __ __ __ / __ __ __ __ _______________________

Name of Participant *Date (DD/MMM/YYYY)* *Signature*

_______________________ __ __ / __ __ __ / __ __ __ __ _______________________

Name of Person taking Consent *Date (DD/MMM/YYYY)* *Signature*

**1 copy for participant; Original for participant CRF; extra copy for medical notes [NHS sites]*
