# Supplementary material for: Phase II multicentre double-blind randomised controlled trial of a Bivalent VaccInation against Salmonella Typhi and Paratyphi A (BiVISTA) using a controlled human infection model of paratyphoid A infection: study protocol
Source: BMJ Open. 2026 Jan 27;16(1):e107608. doi: 10.1136/bmjopen-2025-107608 (PMC12853460; doi:10.1136/bmjopen-2025-107608)
Supplement: online supplemental file 2 [file bmjopen-16-1-s002.docx]

# Supplementary Material

Table of Contents

[Supplementary Material 1](#_Toc1966660690)

[BiVISTA List of Investigators and participating sites 1](#_Toc1846721643)

[List of Inclusion and Exclusion Criteria 2](#_Toc614590235)

[Inclusion Criteria: 2](#_Toc1172885831)

[Exclusion Criteria 3](#_Toc1096566703)

[Figure S1. Screening 7](#_Toc1193711177)

[Figure S2. Group holding rules 9](#_Toc1696881070)

[Box S1: Criteria for Commencing antibiotics 10](#_Toc395775177)

[Box S2: Antibiotics used in the study: second and third lines are only used in case of intolerance to first line course 11](#_Toc738948227)

[Table S1: Complete list of objectives and outcomes 11](#_Toc207648819)

[Table S2: Severity grading of vital signs 13](#_Toc1107764400)

[Table S3: Local Solicited symptoms post-vaccination and Severity Grading 14](#_Toc1921707898)

[Table S4: Systemic Solicited symptoms post-vaccination and Severity Grading 15](#_Toc1657919717)

[Table S5: Solicited symptoms post-challenge and Severity Grading 16](#_Toc1007429248)

[Table S6: Severity Grading of Laboratory Adverse Events 17](#_Toc1144308123)

[Table S7: Sample collection during trial 18](#_Toc1574787980)

[Table S8: Concomitant medications for symptomatic control during challenge phase 19](#_Toc1968663108)

[Table S9: Adverse Events Definitions 20](#_Toc197692929)

## BiVISTA List of Investigators and participating sites

| **Investigator** | **Study centre (participating sites)** |
| --- | --- |
| Professor Andrew J Pollard  Chief Investigator | Oxford Vaccine Group, University of Oxford |
| Professor Xinxue Liu  Lead Statistician | Oxford Vaccine Group, University of Oxford |
| Professor Maheshi Ramasamy  Principal Investigator | Oxford Vaccine Group, University of Oxford |
| Professor Brian Angus  Principal Investigator | Oxford Vaccine Group, University of Oxford |
| Professor Saul Faust  Principal Investigator | University Hospital Southampton NHS Foundation Trust |
| Dr Christopher Green  Principal Investigator | NIHR Birmingham Clinical Research Facility, University Hospitals Birmingham NHS Foundation Trust |
| Dr Rajeka Lazarus  Principal Investigator | University Hospitals Bristol and Weston NHS Foundation Trust |
| Dr Ruth Payne  Principal Investigator | Royal Hallamshire Hospital, Sheffield Teaching Hospitals NHS Foundation Trust |
| Dr Patrick Lillie  Principal Investigator | Hull University Teaching Hospitals NHS Trust & Hull York Medical School |
| Dr Alison Jane Uriel  Principal Investigator | North Manchester General Hospital, Manchester University NHS Foundation Trust |
| Dr David Turner  Principal Investigator | University of Nottingham and University of Nottingham Health Service, and Nottingham University Hospitals NHS Trust |
| Dr Andrea Collins  Principal Investigator | Liverpool School of Tropical Medicine, Royal Liverpool and Broadgreen University Hospital Trust |
| Dr Catherine Cosgove  Principal Investigator | St George’s Hospital London |
| Matthijs Backx  Principal Investigator | University Hospital of Wales, Public Health Wales NHS Trust, Cardiff & Vale University Health Board |
| Dr Christopher Duncan  Principal Investigator | Newcastle University Medical School and The Newcastle Upon Tyne Hospitals NHS Foundation Trust |
| Dr Tommy Rampling  Principal Investigator | University College London Hospitals |

## List of Inclusion and Exclusion Criteria

### Inclusion Criteria:

Participants must satisfy all the following criteria to be considered eligible for the study:

1. Willing and able to give informed consent for participation in the study.
2. Aged between 18 and 55 years, inclusive, at time of vaccination.
3. In good health as determined by medical history, physical examination and clinical judgement of the study team.
4. Willing to be available at designated site for all required appointments.
5. Agree (in the study team’s opinion) to comply with all study requirements, including capacity to adhere to good personal hygiene and infection control precautions.
6. Agree to allow study staff to contact their GP to access the participant’s vaccination records, medical history and have their opinion solicited as to the participant’s appropriateness for inclusion.
7. Agree to allow study staff to access NHS health records (medical and vaccination history) as required for study purposes.
8. Agree to allow their GP (and/or Consultant if appropriate), to be notified of participation in the study
9. Agree to allow national public health agency to be informed of their participation in the study.
10. Agree to give their close household contacts written information about the participants’ involvement in the study and offering them voluntary screening for *S.* Paratyphi A carriage.
11. Agree to have a 24-hour contact with study staff during the four weeks post challenge and are able to ensure that they are contactable by mobile phone for the duration of the vaccination and challenge period until antibiotic completion.
12. Have internet access to allow completion of the e-diary and real-time safety monitoring.
13. Agree to avoid antipyretic/anti-inflammatory treatment from challenge until advised by a study doctor or until 14 days after challenge.
14. Agree to refrain from donating blood for the duration of the study.
15. Agree to provide their National Insurance/Passport number for the purposes of TOPS registration and for payment of reimbursement expenses.
16. Agree to not receive any inactivated vaccine within 7 days before and after vaccination and 7 before and 21 days after challenge.
17. Agree to not receive any live vaccine or vaccine containing DT or TT within 28 days before vaccination to 28 days after challenge.
18. For participants of childbearing potential: willing to ensure that they or their partner use effective contraception 30 days prior to vaccination and continue to do so until clearance is confirmed.

### Exclusion Criteria

The participant will not be eligible if any of the following apply:

1. History of significant organ/system disease that could interfere with study conduct or completion, in the opinion of the study team. Including, for example, but not restricted to:
2. Cardiovascular disease.
3. Respiratory disease.
4. Haematological disease^[[1]](#footnote-1)^
5. Endocrine disorders.
6. Renal or bladder disease, including history of renal calculi.
7. Biliary tract disease, including biliary colic, asymptomatic gallstones or polyps or previous cholecystectomy.
8. Gastro-intestinal disease including chronic diarrhoea, inflammatory bowel disease, irritable bowel syndrome or diseases requiring use of regular antacids, H2-receptor antagonists, proton pump inhibitors, laxatives or prokinetic agents.
9. Neurological disease.
10. Metabolic disease.
11. Psychiatric illness requiring hospitalisation, or history of schizophrenia and maniac depressive psychosis.
12. Known or suspected drug abuse.
13. Known or suspected alcohol misuse.
14. Infectious disease.
15. Coagulation disorders.
16. Have any known or suspected impairment of immune function, alteration of immune function or prior immune exposure that may alter immune function resulting from, for example:

- Congenital or acquired immunodeficiency, including IgA deficiency.
- History of auto-immune disease.
- Human Immunodeficiency Virus or symptoms/signs suggestive of an HIV-associated condition.
- Receipt of immunosuppressive therapy such as anti-cancer chemotherapy or radiation therapy within the preceding 12 months or long-term systemic corticosteroid therapy.

1. Receipt of immunoglobulin or any blood product transfusion within 3 months of study vaccination.
2. History of cancer (except squamous cell or basal cell carcinoma of the skin and cervical carcinoma in situ).
3. HLA-B27 positive.
4. Moderate or severe depression or anxiety as classified by the Hospital Anxiety and Depression Score at screening or challenge that is deemed clinically significant by the study doctors.
5. Weight less than 50 kg.
6. Presence of implants or prosthetic material.
7. Taking long-term medication (e.g., analgesia, anti-inflammatories or antibiotics) that may affect symptom reporting or interpretation of the study results or that may interact with antibiotics used for treatment of paratyphoid A (in particular drugs that could prolong corrected QT interval).
8. Contraindication to fluoroquinolones, macrolide antibiotics, co-trimoxazole or ceftriaxone.
9. Family history of aneurysmal disease.
10. Participants who are pregnant, lactating or who are unwilling to ensure that they or their partner use effective contraception 30 days prior to vaccination and continue to do so until three negative stool samples have been obtained after completion of antibiotic treatment.
11. Full-time, part-time or voluntary occupations involving the below (unless willing to avoid work from challenge day until demonstrated not to be infected with *S.* Paratyphi A by clearance samples in accordance with guidance from national public health agency and willing to allow study staff to inform their employer):
12. Direct contact with young children (defined as those attending pre-school groups or nursery or aged under 2 years).
13. Direct contact with highly susceptible patients or persons in whom paratyphoid A infection would have particularly serious consequences.
14. Commercial food handling (involving preparing or serving unwrapped foods not subjected to further heating).
15. Close household contact with:
    1. Young children (defined as those attending pre-school groups, nursery or those aged less than 2 years)
    2. Individuals who are immunocompromised (including pregnancy).
16. Scheduled elective surgery or other procedures requiring general anaesthesia during the study period.
17. Participation in another research study involving an investigational product that might affect risk of paratyphoid A infection or compromise the integrity of the study within the 30 days prior to study vaccination (e.g., significant volumes of blood already taken in previous study), including an interval between trials of a minimum of 5 half-lives of the investigational product’s last administration; or plan to enrol in another research study during the follow-up study period.
18. Detection of any abnormal results from screening investigations (at the clinical discretion of the study team).
19. Having been resident in an enteric fever endemic country for 6 months or more.
20. Previous diagnosis with laboratory-confirmed typhoid or paratyphoid infection or a diagnosis compatible with enteric fever.
21. Participation in previous typhoid or paratyphoid A challenge studies (with ingestion of challenge agent).
22. Receipt of any typhoid vaccination at any time.
23. Prolonged corrected QT interval (>450 milliseconds) or significant clinical abnormality on ECG.
24. Presence of gallbladder abnormalities such as stones/calculi or polyps, as seen on ultrasound.
25. Significant blood donation or planned blood donation prior to study vaccination.
26. Known serious reactions, allergy, hypersensitivity or any life-threatening reaction to any of the vaccine components, including reactions to previous receipt of tetanus or diphtheria containing vaccines.
27. Evidence of HIV, Hepatitis B or Hepatitis C infection.
28. Inability to comply with any of the study requirements (at the discretion of the study staff and the participant’s General Practitioner).
29. Any other social, psychological or health issues which, in the opinion of the study staff, may
    1. Put the participant or their contacts at risk because of participation in the study.
    2. Adversely affect the interpretation of the primary endpoint data.
    3. Impair the participant’s ability to participate in the study.

#### **Temporary Exclusion at Vaccination**

Participants will be temporarily excluded from receiving vaccination if presenting at a vaccination visit with the following:

1. Significant infection within the previous 5 days (as per the clinical discretion of the study team)
2. Use of antipyretics in the 24 hours prior to vaccination.
3. Any systemic corticosteroid (or equivalent) treatment in the previous 14 days, or for more than seven consecutive days within the past 3 months).
4. History of systemic antibiotic therapy during the previous 5 days for short-acting antibiotics and 15 days for long-acting antibiotics.
5. Receipt of a live vaccine or DT, TT containing vaccines within 4 weeks prior to the vaccination or an inactivated vaccine within 7 days prior to vaccination.
6. Plan to receive any vaccine other than the study vaccine within 7 days following vaccination.
7. Unavailable for post-vaccination visits, and challenge visit as outlined in study procedures table.
8. Any current illness considered, in the opinion of the investigator, requiring of further time/investigation to resolve or stabilise prior to a dose of vaccine being administered.

If the temporary exclusion does not result in the participant becoming ineligible, then the vaccine visit can be rescheduled.

#### **Temporary Exclusion at Challenge**

Participants will be temporarily excluded from challenge if presenting at the challenge visit with the following:

1. Acute or acute-on-chronic infection within the previous 5 days, that is considered clinically significant by the Investigator
2. History of any systemic antibiotic therapy during the previous 5 days for short-acting antibiotics and 15 days for long-acting antibiotics.
3. Any systemic corticosteroid (or equivalent) treatment in the previous 14 days, or for more than seven consecutive days within the past 3 months.
4. Therapy with antacids, proton pump inhibitors or H2-receptor antagonists or prokinetic agents within 24 hours prior to challenge.
5. Occurrence of a laboratory adverse event such as anaemia, which in the opinion of the Investigator, requires further time and/or investigation to resolve or stabilize prior to challenge being administered.
6. Plan to receive any vaccine within 21 days following challenge.

If the temporary exclusion does not result in the participant becoming ineligible, then the challenge visit can be rescheduled.

## Figure S1. Screening

A. Screening process

B. Screening tests

## Figure S2. Group holding rules

## Box S1: Criteria for Commencing antibiotics

| Antibiotics are commenced if ANY of the following apply |
| --- |
| Any participant meeting the definition of paratyphoid A infection |
| Any participant with 3 or more of the following symptoms *severe enough to interfere with all normal activity* (after discussion with CI or PI):   - Headache - Malaise - Anorexia - Abdominal pain - Nausea/vomiting - Myalgia - Arthralgia - Cough - Rash - Diarrhoea - Constipation |
| Any participant who has not received antibiotics by day 14 post-challenge |
| Any participant in whom antibiotic use is felt to be clinically necessary (as decided by a medically qualified study doctor) |
| Participants withdrawn after challenge administration, including participants who vomit within 90 minutes of challenge administration |
| Relapse of Paratyphoid A infection |
| Presence of convalescent shedding of *Salmonella* Paratyphi A in stools (at least one week after initial antibiotic treatment) |

## Box S2: Antibiotics used in the study: second and third lines are only used in case of intolerance to first line course

| **First-line of treatment** | - Oral ciprofloxacin 750 mg twice daily from the 1^st^ to the 4^th^ day (total of 8 doses) followed by oral azithromycin at 1,000 mg on the 5^th^ day and 500 mg once daily from the 6^th^ to the 14^th^ day |
| --- | --- |
| **Second-line of treatment** | - Oral trimethoprim/sulfamethoxazole 160/800 mg twice daily for 14 days or - Oral azithromycin 1g single dose followed by 500 mg daily for 13 days or - Ciprofloxacin 750 mg twice daily for 14 days |
| **Third-line of treatment** | - Oral amoxicillin 500 mg three times a day for 14 days |

## Table S1: Complete list of objectives and outcomes

| **Objectives** | **Outcome Measures** |
| --- | --- |
| **Primary** | |
| To evaluate the efficacy of SII-TCV(B) against *S*. Paratyphi A compared with a licensed Vi polysaccharide vaccine in a healthy adult paratyphoid A challenge model | Proportion of participants developing *S.* Paratyphi A infection within 14 days following challenge |
| To evaluate the non-inferiority of the immune response to the *Salmonella* Typhi component of SII-TCV(B) compared with the licensed Vi Polysaccharide vaccine | Geometric Mean Concentration of *S.* Typhi Vi antigen-specific IgG at day 28 following vaccination |
| **Secondary** | |
| To evaluate the safety and tolerability of SII-TCV(B) compared with a licensed Vi polysaccharide vaccine | Occurrence of:  - local solicited events in 7 days following vaccination  - systemic solicited events in 7 days following vaccination  - unsolicited events in 28 days following vaccination  - serious adverse events following vaccination throughout study participation |
| To evaluate the immunogenicity of SII-TCV(B) compared with a licensed Vi polysaccharide vaccine | 1. Quantification of *S.* Paratyphi A antigen-specific antibodies at 28 days following vaccination: GMCs, GMFR and seroconversion (four folds rise to pre-vaccination) for:  - Anti-LPS IgG, IgA and IgM  - SBA titres  2. Quantification of *S.* Typhi antigen-specific antibodies at 28 days following vaccination: GMCs, GMFR and seroconversion (four folds rise to pre-vaccination) for Anti-Vi IgG and IgA |
| **Exploratory** | |
| To evaluate the effect of SII-TCV(B) on the clinical course of *S.* Paratyphi A infection | 1. Time to diagnosis  2. Time to symptom onset  3. Symptom severity  4. Duration of illness  5. Pattern of stool shedding  6. Inflammatory response as measured by C-reactive protein |
| To compare the host immune response following *S*. Paratyphi A challenge following vaccination with SII-TCV(B) or a licensed Vi polysaccharide vaccine. | Quantification of *S.* Paratyphi A antigen-specific antibodies at 28-days following challenge: GMCs, and GMFR (to pre-challenge) for:  - Anti-LPS IgG, IgA and IgM  - SBA titres |
| To investigate additional humoral and cellular immunogenicity of paratyphoid A component following vaccination with SII-TCV(B) | Quantification of immunological response data post-vaccination including:  - Quantification of *S.* Paratyphi A antigen-specific IgG, IgA and IgM antibodies at 7 and 14 days following vaccination  - Carrier protein specific cell-mediated responses (including antigen specific cell frequencies, description of lymphocyte populations, and T cell repertoire)  - B cell responses to LPS  - Functional responses to LPS  - Mucosal responses |
| To investigate immunological correlates of protection for *S*. Paratyphi A infection | To determine if antibody titres for paratyphoid A component correlate with protection against development of paratyphoid A infection. |
| To investigate the microbiological response following challenge with *S.* Paratyphi A | Assessment of the quantitative level of bacteraemia in diagnosed participants |
| To investigate recruitment methods and reasons for participant exclusions from paratyphoid A challenge models | Analysis of recruitment numbers, including:  - Number of positive and negative responses to different recruitment techniques;  - Number of participants excluded prior to attending screening visits and reasons for exclusion;  - Number of participants attending for screening visits and reasons for exclusion |
| To explore the variation in genomic response to vaccination with SII-TCV(B), or control and subsequent *S.* Paratyphi A challenge | Laboratory and high-throughput assays to measure gene expression and protein translation at baseline, post-vaccination, post-challenge and at diagnosis time points. |
| To explore molecular changes and metagenomics occurring after vaccination, challenge and during acute infection | Application of techniques such as proteomics, metabolomics, epigenetics and metagenomics to samples from baseline, post-vaccination and post-challenge timepoints. |

## Table S2: Severity grading of vital signs

| **Observation** | **Grade 1** | **Grade 2** | **Grade 3** | **Grade 4*** |
| --- | --- | --- | --- | --- |
| **Oral temperature (°C)** | 37.6-38.0 | 38.1-39.0 | 39.1-40.0 | > 40.0 |
| **Tachycardia (beats/min)** | 101-115 | 116-130 | > 130 | Presentation to A&E or Hospitalisation for arrhythmia |
| **Bradycardia (beats/min) **** | 50-54 | 45-49 | < 45 | Presentation to A&E or Hospitalisation for arrhythmia |
| **Systolic hypertension (mmHg)** | 141-150 | 151-155 | > 155 | Presentation to A&E or Hospitalisation for malignant hypertension |
| **Diastolic hypertension (mmHg)** | 91-95 | 96-100 | > 100 | Presentation to A&E or Hospitalisation for malignant hypertension |
| **Systolic hypotension (mmHg)** | 85-89 | 80-84 | < 80 | Presentation to A&E or Hospitalisation for hypotensive shock |
| *Grade 4 adverse events, when causing hospitalisation, should be reported as SAE (serious adverse event), as defined on Table S9  ** Clinical judgement should be used when characterizing bradycardia in healthy subjects, in particular young conditioned participants and athletes. | | | | |

## Table S3: Local Solicited symptoms post-vaccination and Severity Grading

| **Adverse Event** | **Grade** | **Intensity** |
| --- | --- | --- |
| **Pain at injection site** | 1 | Pain that is easily tolerated, not interfering with daily activity |
|  | 2 | Pain that requires repeated (more than once) use of non-narcotic pain-reliever medication or interferes with daily activity |
|  | 3 | Use of narcotic pain-reliever (such as codeine) or pain that prevents any daily activity |
|  | 4* | Presentation to A&E or Hospitalisation for pain management |
| **Tenderness at injection site** | 1 | Mild discomfort to touch |
|  | 2 | Discomfort with movement |
|  | 3 | Significant discomfort at rest |
|  | 4* | Presentation to A&E or Hospitalisation for pain management |
| **Injection site redness**** | 1 | 2.5 - 5 cm |
|  | 2 | 5.1 - 10 cm |
|  | 3 | >10 cm |
|  | 4* | Exfoliative dermatitis (as graded by study doctor) |
| **Injection site swelling/ induration** | 1 | 2.5 – 5 cm |
|  | 2 | 5.1 - 10 cm |
|  | 3 | >10 cm |
|  | 4* | Necrosis (as graded by study doctor) |
| *Grade 4 adverse events, when causing hospitalisation or when life-threatening, should be reported as SAE (serious adverse event), as defined on Table S9  ****Erythema (redness) and swelling/induration ≤2.5cm is an expected consequence of skin puncture and will therefore not be considered an adverse event** | | |

## Table S4: Systemic Solicited symptoms post-vaccination and Severity Grading

|  | **1** | **2** | **3** | **4*** |
| --- | --- | --- | --- | --- |
| **Headache** | Present but no interference with activity | Some interference with activity | Significant; any use of codeine phosphate or prevents any normal daily activity | Presentation to A&E or Hospitalisation |
| **Generally unwell or fatigue** | Present but no interference with activity | Some interference with activity | Significant; prevents any normal daily activity | Presentation to A&E or Hospitalisation |
| **Muscle pain** | Present but no interference with activity | Some interference with activity | Significant; prevents any normal daily activity | Presentation to A&E or Hospitalisation |
| **Joint pain** | Present but no interference with activity | Some interference with activity | Significant; prevents any normal daily activity | Presentation to A&E or Hospitalisation |
| **Nausea/vomiting** | Present but no interference with activity or 1 – 2 episodes in 24 hours | Some interference with activity or more than 2 episodes in 24 hours | Significant; prevents any normal daily activity or requires intravenous hydration | Hospitalisation |
| **Eating less than usual or loss of appetite** | Eat less than normal for 1-2 meals | Miss 1-2 meals completely | Miss all meals in the 24 hrs | Presentation to A&E or Hospitalisation |

## Table S5: Solicited symptoms post-challenge and Severity Grading

|  | **1** | **2** | **3** | **4*** |
| --- | --- | --- | --- | --- |
| **Headache** | Present but no interference with activity | Some interference with activity | Significant; any use of codeine phosphate or prevents any normal daily activity | Presentation to A&E or Hospitalisation |
| **Generally unwell or fatigue** | Present but no interference with activity | Some interference with activity | Significant; prevents any normal daily activity | Presentation to A&E or Hospitalisation |
| **Eating less than usual or loss of appetite** | Eat less than normal for 1-2 meals | Miss 1-2 meals completely | Miss all meals in the 24 hrs | Presentation to A&E or Hospitalisation |
| **Abdominal/ stomach pain** | Present but no interference with activity | Some interference with activity | Significant; any use of codeine phosphate or prevents any normal daily activity | Presentation to A&E or Hospitalisation |
| **Nausea/vomiting** | Present but no interference with activity or 1 – 2 episodes in 24 hours | Some interference with activity or more than 2 episodes in 24 hours | Significant; prevents any normal daily activity or requires outpatient/day-hospital intravenous hydration | Hospitalisation |
| **Muscle pain** | Present but no interference with activity | Some interference with activity | Significant; any use of codeine phosphate or prevents any normal daily activity | Presentation to A&E or Hospitalisation |
| **Joint pain** | Present but no interference with activity | Some interference with activity | Significant; any use of codeine phosphate or prevents any normal daily activity | Presentation to A&E or Hospitalisation |
| **Cough** | Present but no interference with activity | Some interference with activity | Significant; any use of codeine phosphate or prevents any normal daily activity | Presentation to A&E or Hospitalisation |
| **Diarrhoea** | 3-4 loose stools in 24 hrs | 5-6 loose stools in 24 hrs | 7 or more loose stools in 24 hrs or requires outpatient/day-hospital intravenous hydration | Hospitalisation |
| **Constipation** | Present but no interference with activity | Some interference with activity or use of laxatives | Significant; prevents daily activity | Presentation to A&E or Hospitalisation |
| **Rash **** | YES/ NO | Describe where, colour, size (mm): | | |
| **New low mood or  worsening mood** | YES/ NO | Please describe what the change is: | | |
| ** We may take pictures of the rash for the records, upon participant verbal consent. | | | | |

## Table S6: Severity Grading of Laboratory Adverse Events

| **Parameter** | **Grade 1** | **Grade 2** | **Grade 3** | **Grade 4*** |
| --- | --- | --- | --- | --- |
| **Haemoglobin: decrease from baseline value (g/l)** | 10 – 15 | 16-20 | 21-50 | > 50 |
| **White cell count: elevated (10^9^/L)** | 11.01–15 | 15.01–20 | 20.01-25 | > 25 |
| **White cell count: depressed (10^9^/L)** | 2.5-3.5 | 1.5-2.49 | 1.00-1.49 | < 1.00 |
| **Neutrophil count (10^9^/L)** | 1.5-2.0 | 1.0-1.49 | 0.50-0.990 | < 0.5 |
| **Platelets: decreased (10^9^/L)** | 125-140 | 100-124 | 25-99 | < 25 |
| **Sodium: hyponatraemia (mmol/L)** | 132–134 | 130–131 | 125-129 | < 125 |
| **Sodium: hypernatraemia (mmol/L)** | 146 | 147 | 148-150 | > 150 |
| **Potassium: hyperkalaemia (mmol/L)** | 5.4-5.5 | 5.6-5.7 | 5.8-5.9 | > 6.0 |
| **Potassium: hypokalaemia (mmol/L)** | 3.3–3.4 | 3.1–3.2 | 3.0 | < 3.0 |
| **Urea (mmol/L)** | 8.2–9.3 | 9.4–11.0 | > 11.0 | Requires dialysis |
| **Creatinine (μmol/L)** | 132-150 | 151-177 | 178-221 | > 221 or requires dialysis |
| **ALT (IU/L)** | 51-118 | 119-234 | 235-399 | > 400 |
| **Bilirubin, with increase in LFTs (μmol/L)** | 23-26 | 27-31 | 32-37 | > 37 |
| **Bilirubin, with normal LFTs (μmol/L)** | 23-31 | 32-42 | 43-63 | > 63 |
| **Alkaline phosphatase (IU/L)** | 146-277 | 278-409 | 410-1700 | > 1700 |
| **Albumin: hypoalbuminaemia (g/L)** | 28–31 | 25–27 | < 25 | Not applicable |
| **C-reactive protein** | 11-30.9 | 31-100.9 | 101-200 | > 200 |
| *Grade 4 laboratory adverse events may be life-threatening. If that is the case, the adverse event should be reported as SAE (serious adverse event)  NB: During challenge phase, in diagnosed participants, any of the following laboratory results will not be reported as AE:  a. Haemoglobin that would be reported as grade 1 or 2;  b. White cell count (elevated or depressed) that would otherwise be reported as grade 1 or 2;  c. ALT or AST that would otherwise be reported as grade 1 or 2;  d. C-reactive protein that would otherwise be reported as grade 1 or 2 or 3 or 4;  e. Albumin (hypoalbuminaemia) that would otherwise be reported as grade 1 or 2. | | | | |

## Table S7: Sample collection during trial

|  | Visit day | Haematology  (blood)^1^ | Biochemistry  (blood) ^1^ | Blood culture^1^ | Serum samples  (blood) | Other research samples (blood) | Saliva samples^2^ | Stool samples^3^ | Urine (for pregnancy) |
| --- | --- | --- | --- | --- | --- | --- | --- | --- | --- |
| Vaccination phase | D-28 | x | x |  | x | x | x | x | x |
|  | D-21 and D-14 | x | x |  | x | x | x | x^2^ |  |
|  | Post-vaccine immunology (D-2a) **^5^** |  |  |  | x |  |  |  |  |
| Pre-challenge | Pre-challenge  (D-2 and D0) ^4^ | x | x |  |  | x | x | x | x |
| Challenge Phase (before diagnosis or if no diagnosis is made) | D1 |  |  | x |  | x |  | x |  |
|  | D3, D5, D9, D11, D13 |  |  | x |  |  |  | x |  |
|  | D2, D4, D6, D8, D12 | x | x | x |  |  |  | x |  |
|  | D7 |  |  | x | x | x |  | x |  |
|  | D10 | x | x | x |  | x |  | x |  |
|  | D14 | x | x | x |  | x |  | x | x |
| If Paratyphoid A diagnosis made | PD | x | x | x |  | x |  | x | x |
|  | PD+12 and/or PD+24 ^6^ | x | x | x |  | x |  | x |  |
|  | PD+48, PD+72 | x | x | x |  |  |  | x |  |
|  | PD+96 | x | x | x |  | x |  | x |  |
|  | D14PD | x | x |  |  |  |  | x |  |
| Acute and Long-Term Follow-Up | D28, D90 and D180 | x | x |  | x | x |  | x |  |
| ^[1]^- These samples can be collected at any point to ensure participant’s safety. ^[2]^- Only collected for a small subset of participants. ^[3]^- Stool samples are also collected to confirm faecal clearance of *S.* Paratyphi A at least 1 week after completion of a full course of antibiotics, until 3 successive stool samples are culture negative for *S.* Paratyphi A. ^[4]^- Pre-challenge activities may occur between 0 and 48 hours before D0, depending on parent site capacity for activities to happen on challenge day itself. All pre-challenge activities should be performed by the participant’s parent site prior to challenge day. ^[5]^- In almost all cases the Post-Vaccine Immunology visit will occur at the same time as Pre-challenge visit, however if challenge is delayed beyond the window of 26-35 days post vaccination, these visits would happen on separate days to allow for immunology bloods to be collected in time. ^[6]^- PD+12 is an optional safety visit. PD+12 and PD+24 visits do not need to both occur. Completion of at least one of them is required, and the other one may also be required for safety reasons. This is to be decided at the clinical discretion of the investigator. | | | | | | | | | |

## Table S8: Concomitant medications for symptomatic control during challenge phase

| Drug | Indication | Dose | Route | Frequency |
| --- | --- | --- | --- | --- |
| Paracetamol | Fever and discomfort (**after antibiotic therapy started**) | 500 mg – 1 Gram | Oral | PRN, maximum four times a day |
| Codeine | Pain including headache | 15-60 mg | Oral | PRN (maximum 240 mg over 24 hours) |
| Senna | Constipation | 1-4 tablets | Oral | PRN daily |
| Cyclizine | Nausea and/or vomiting | 50 mg | Oral | PRN (maximum 150 mg over 24 hours) |
| Chlorpheniramine | Allergy | 4 mg | Oral | PRN three to four times daily (maximum 24 mg over 24 hours) |
| Oral rehydration salts | Dehydration, vomiting or diarrhoea | 1-2 sachets | Oral | PRN daily |
| Sando-K | Hypokalaemia | 2-4 tablets | Oral | PRN, up to three times a day, dependent, on potassium deficit |
| PRN: *pro re nata*, i.e., as required | | | | |

## Table S9: Adverse Events Definitions

| Adverse Event (AE) | Any untoward medical occurrence in a participant to whom a medicinal product has been administered, including occurrences which are not necessarily caused by or related to that product. |
| --- | --- |
| Adverse Event Related to Challenge for Special Consideration (AERC) | An adverse event following challenge that is of scientific and medical concern related to the administration of challenge, for which ongoing monitoring and rapid communication to the safety committee and/or Sponsor delegate may be appropriate. |
| Adverse Reaction (AR) | An untoward and unintended response in a participant to an investigational product which is related to any dose administered to that participant.  The phrase “response to an investigational medicinal product” means that a causal relationship between the IMP and an AE is at least a reasonable possibility, i.e., the relationship cannot be ruled out.  All cases judged by either the reporting medically qualified professional or the Sponsor delegate as having a reasonable suspected causal relationship to the trial medication qualify as adverse reactions. |
| Serious Adverse Event (SAE) | A serious adverse event (SAE) is any untoward medical occurrence that:   - results in death - is life-threatening (i.e., the participant was, in the view of the investigator, at immediate risk of death from the event that occurred). This does not include an AE that, if it occurred in a more serious form, might have caused death. - requires inpatient hospitalisation or prolongation of existing hospitalisation. Hospitalisation (including inpatient or outpatient hospitalisation for an elective procedure) for a pre-existing condition that has not worsened unexpectedly does not constitute a serious AE. - results in persistent or significant disability/incapacity - congenital anomaly or birth defect - an ‘important medical event’ (that may not cause death, be life threatening or require hospitalisation) may also be considered a serious adverse event when, based upon appropriate medical judgement, the event may jeopardise the participant and may require medical or surgical intervention to prevent one of the outcomes listed above. |
| Serious Adverse Reaction (SAR) | An adverse event that is both serious and, in the opinion of the reporting Investigator, believed to be related to an IMP, based on the information provided. |
| Suspected Unexpected Serious Adverse Reaction (SUSAR) | A serious adverse reaction, the nature and severity of which is not consistent with the Reference Safety Information for the medicinal product in question set out:   - in the case of a product with a marketing authorisation, in the approved summary of product characteristics (SmPC) for that product - in the case of any other investigational medicinal product, in the approved investigator’s brochure (IB) relating to the trial in question.   **For the purposes of this study, all SARs will be considered SUSARs.** |

1. This includes anaemia. The acceptable lower limits for haemoglobin concentration are 125 g/L for female participants and 135 g/L for male participants (Guidelines for the Blood Transfusion Services in the UK, 8th edition, updated 4^th^ September 2023 < https://www.transfusionguidelines.org/red-book> Accessed 5th September 2023). [↑](#footnote-ref-1)
