# Supplementary material for: Phase II multicentre double-blind randomised controlled trial of a Bivalent VaccInation against Salmonella Typhi and Paratyphi A (BiVISTA) using a controlled human infection model of paratyphoid A infection: study protocol
Source: BMJ Open. 2026 Jan 27;16(1):e107608. doi: 10.1136/bmjopen-2025-107608 (PMC12853460; doi:10.1136/bmjopen-2025-107608)
Supplement: Supplementary file 3 [file bmjopen-2025-107608.reviewer_comments.pdf]

PEER REVIEW HISTORY

BMJ Open publishes all reviews undertaken for accepted manuscripts. Reviewers are asked to complete a checklist review form and are provided with free text boxes to elaborate on their assessment. These free text comments are reproduced below.

ARTICLE DETAILS

Title (Provisional)

Study protocol for BiVISTA (Bivalent Vaccination against Salmonella Typhi and Paratyphi A): a phase II multicentre double-blind randomised controlled trial using a controlled human infection model of paratyphoid A infection

Authors

Paganotti Vicentine, Margarete; McCann, Naina; Hennigan, Oisin; Maria, Noshi; Juarez Molina, Claudia I; Koleva, Stanislava; Islam, Mohammod K; Jones, Elizabeth; Flaxman, Amy; Day, Nicole; MacDonald, Allison; Adnan, Mehreen; Singh, Nisha; Vernon, Sophie; Wilson, Eleanor; Potey, Anirudha Vyankatesh; Dharmadhikari, Abhijeet; Gaidhane, Sanket; Kulkarni , Prasad S; Robinson, Hannah; Aley, Parvinder; Kim, Young Chan; Angus, Brian; Liu , Xinxue; Ramasamy, Maheshi Nirmala N.; Pollard, Andrew J

VERSION 1 - REVIEW

**Reviewer**	**1**
**Name**	**Koley, Hemanta**
**Affiliation**	**ICMR - National Institute of Cholera and Enteric Diseases**
**Date**	**08-Oct-2025**
**COI**	**None**

The manuscript entitled “Study protocol for BiVISTA (Bivalent Vaccination against Salmonella Typhi and Paratyphi A): a 4 phase II multicentre double-blind randomised controlled trial using a controlled human 5 infection model of paratyphoid A infection” provides a well-articulated and scientifically robust proposal for evaluating the efficacy of a bivalent vaccine against Salmonella enterica serovars Typhi and Paratyphi A using a Controlled Human Infection Model (CHIM). The rationale is strong, addressing an urgent global health need for broader enteric fever prevention, particularly in South Asia where both pathogens are co-endemic. The design aligns well with WHO PDVAC recommendations, and the integration of immunogenicity bridging data with a planned Phase III trial in India enhances the translational and regulatory potential of the study.

The discussion effectively acknowledges that CHIM participants differ from target endemic populations in age and prior exposure. However, it would benefit from elaboration on how these differences could influence immunological responses and the interpretation of efficacy data. Inclusion of additional details on immunological correlates of protection for S. Paratyphi A and ethical considerations—such as participant safety, early antibiotic rescue, and informed consent—would further strengthen the manuscript's transparency.

Overall, this study is well-conceived, methodologically sound, and of high public health importance. If successful, it could significantly advance the development and deployment of bivalent enteric fever vaccines, contributing to disease burden reduction and antimicrobial resistance mitigation in endemic regions.

**Reviewer**	**2**
**Name**	**White, Aaron P**
**Affiliation**	**University of Saskatchewan**
**Date**	**17-Nov-2025**
**COI**	**None**

BMJ open #2025-10760B

Well-designed clinical trial plan, with good methods and tracking in place.

In general, I see no major problems with the author's plan, but I do have a few questions.

1. Are the clinical manifestations or paratyphoid infections different than Typhoid fever? What will be tracked? This could be explained better.

a. I guess from reading that everything comes back to blood culture of Salmonella serovar Paratyphi A

b. What criteria will be used to ensure that it is the same strain that has been recovered? PCR and sequencing? Whole genome sequencing?

2. Under recruitment and eligibility, how you will address sex differences? I think it's important to state how this will be matched, as well as the age of participants

a. The Figure with "Vaccination phase ⍰ Challenge phase ⍰ Follow-up phase" shows equal men and women, but this should be explicitly stated somewhere

3. How will you track antibiotic use, once the patients start taking them at Day 14?

a. More of an ethical consideration than anything that will affect the trial

b. Will the antibiotics be administered by Doctors in the trial? Or will the patient be given their doses to take home?

4. Footnotes to Table S2, S3, show the message “Error! Reference source not found.” These should be corrected with the proper reference

---

VERSION 1 - AUTHOR RESPONSE

Reviewer 1 (Dr. Hemanta Koley) comments and responses:
- The discussion effectively acknowledges that CHIM participants differ from target endemic populations in age and prior exposure. However, it would benefit from elaboration on how these differences could influence immunological responses and the interpretation of efficacy data.
The discussion session has been expanded to address anticipated immunological differences between the BiVISTA study participants and the population in the endemic regions, with implications for immunobridging to phase III data in the fifth paragraph of the discussion, page 19.

- Inclusion of additional details on immunological correlates of protection for S. Paratyphi A and ethical considerations—such as participant safety, early antibiotic rescue, and informed consent—would further strengthen the manuscript’s transparency.
Two paragraphs were added to the discussion session expanding on correlates of protection (seventh paragraph, page 20) and on ethical considerations (third paragraph, page 19).

Reviewer 2 (Dr. Aaron P White) comments and responses:
1. Are the clinical manifestations or paratyphoid infections different than Typhoid fever? What will be tracked? This could be explained better.
a. I guess from reading that everything comes back to blood culture of Salmonella serovar Paratyphi A
Many thanks for this question. The clinical manifestations of typhoid and paratyphoid A infections are indistinguishable, but OVG’s paratyphoid CHIM using the NVGH308 strain at the dose of 1 5 x 103 CFU has a milder symptom profile than OVG’s typhoid CHIM using the Quailes strain at the dose of 1 5 x 104 CFU. We have added clarity to the “Safety and Potential Risks” section, subsection of the challenge, first paragraph, page 16.

b. What criteria will be used to ensure that it is the same strain that has been recovered? PCR and sequencing? Whole genome sequencing?
Thank you for this question. Recruited participants are naïve to enteric fever and baseline stool samples are collected to ensure no Salmonella Paratyphi A is present prior to challenge. Since the UK is not endemic for enteric fever, and participants will not travel to endemic countries, we will assume that all Salmonella Paratyphi A recovered from blood or stool culture will be the challenge strain.

2. Under recruitment and eligibility, how you will address sex differences? I think it’s

important to state how this will be matched, as well as the age of participants

a. The Figure with “Vaccination phase Challenge phase Follow-up phase” shows equal men and women, but this should be explicitly stated somewhere

Based on previous paratyphoid CHIM studies, we did not observe any difference in challenge attack rates in male or female populations. Although we are not planning to match sex due to this reason, i.e., exactly 50% male and female participants, the trial management group will actively monitor the gender and age ratio in our study. The eligibility criteria were carefully considered to reduce sex and age disparities in recruitment, but we do acknowledge that there may be a higher rate of male participants due to contraception requirements for female participants. The figure shows equal men and women to illustrate representativeness. We have added one sentence in the recruitment section to clarify that the trial management group will actively monitor the representativeness of our trial population, page 9, line 16.

3. How will you track antibiotic use, once the patients start taking them at Day 14?

a. More of an ethical consideration than anything that will affect the trial

Thank you, this information was added to the manuscript, under the Trial interventions section, antibiotics subsection, on page 12, line 37-38. Participants will be asked to complete the e-diary with the date and time at which each dose was taken for monitoring compliance.

b. Will the antibiotics be administered by Doctors in the trial? Or will the patient be given their doses to take home?

Thank you, this information was added to the manuscript, under the Trial interventions section, antibiotics subsection, on page 12, line 37-38. Participants will be seen taking the first dose of antibiotics and given the rest of the doses to be taken at home.

4. Footnotes to Table S2, S3, show the message “Error! Reference source not found.” These should be corrected with the proper reference

Thank you for pointing this out, these references were corrected.
